# Positive Mental Health, Anxiety and Prenatal Bonding: A Contextual Approach

**DOI:** 10.3390/healthcare13243300

**Published:** 2025-12-16

**Authors:** Laura Xu Ballesteros-Andrés, Raquel Luengo-González, Inmaculada Concepción Rodríguez-Rojo, Montserrat García-Sastre, Daniel Cuesta-Lozano, Jorge-Luis Gómez-González, José Alberto Martínez-Hortelano, Cecilia Peñacoba-Puente

**Affiliations:** 1 Francisco Díaz Mental Health Centre, Príncipe de Asturias Hospital, 28805 Alcalá de Henares, Spain; lauraxuballesteros@gmail.com; 2 Community Care and Social Determinants of Health (CUYDET), Nursing and Physiotherapy Department, Universidad de Alcalá, 28804 Alcalá de Henares, Spain; raquel.luengo@uah.es (R.L.-G.); mmontserrat.garcia@uah.es (M.G.-S.); daniel.cuesta@uah.es (D.C.-L.); 3 Group for Research in Nursing Care, Gregorio Marañón, Health Research Institute (IiSGM), 28009 Madrid, Spain; 4 Center for Cognitive and Computational Neuroscience (C3N), Universidad Complutense, 28040 Madrid, Spain; 5 Nursing and Physiotherapy Department, Universidad de Alcalá, 28804 Alcalá de Henares, Spain; jorgeluis.gomez@uah.es; 6 Faculty of Nursing, University of Castilla-La Mancha, 02006 Albacete, Spain; josealberto.martinez@uclm.es; 7 Psychology Department, Universidad Rey Juan Carlos, 28933 Madrid, Spain; cecilia.penacoba@urjc.es

**Keywords:** positive mental health, prenatal bonding, pregnancy anxiety, perinatal well-being, mediation model

## Abstract

**Background/Objectives:** The establishment of strong prenatal bonding is a key determinant of perinatal well-being, influencing maternal psychological adaptation and infant development. Numerous studies have examined risk factors and psychopathology during pregnancy, but limited research has explored the role of positive psychological constructs, such as positive mental health (PMH). This study aimed to assess whether anxiety mediates the relationship between PMH and the quality of prenatal bonding. **Methods:** A total of 90 pregnant women participated. PMH was assessed using the Abbreviated Positive Mental Health Questionnaire; anxiety using the Hospital Anxiety and Depression Scale; and prenatal bonding using the Prenatal Assessment Scale for Pregnant Women (EVAP). A simple mediation model was tested, with anxiety as a mediator between PMH (predictor) and prenatal bonding (outcome), controlling the analysis for previous miscarriages, relationship stability, high-risk pregnancy, and employment. **Results:** The model revealed partial mediation (F = 16.617, *p* < 0.001). Higher PMH was associated with lower anxiety (B = −0.297, SE = 0.062, *p* < 0.001) and stronger prenatal bonding (B = 0.777, SE = 0.091, *p* < 0.001). Interestingly, anxiety emerged as an adaptive response, which could improve maternal sensitivity and communication with the unborn child (B = 0.316, SE = 0.145, *p* = 0.032). The model explained 56% of the variance in prenatal bonding, even after accounting for relevant covariates. **Conclusions:** These findings underscore the importance of considering contextual and psychosocial factors when assessing the role of emotions such as anxiety during pregnancy. Rather than being inherently maladaptive, anxiety may play a functional role in facilitating maternal engagement with the baby, especially when grounded in PMH. Given the limited research, our findings support the integration of positive psychology frameworks into perinatal health interventions.

## 1. Introduction

The transition to parenthood and the arrival of children are recognized as a maturational crisis that brings about changes and adjustments within the family, especially for pregnant women. Pregnancy involves significant physiological and psychosocial transition that increases women’s emotional and psychological vulnerability, making them more susceptible to anxiety and depression [1]. Pregnant women often experience uncertainty about their maternal abilities and substantial changes in their identity and expectations about their role, which can increase psychological stress and predispose them to elevated levels of mental health symptoms [2,3,4].

A mental health deficit negatively affects maternal and fetal health, contributing to a higher obstetric risk during childbirth. It can also impact the baby’s development, leading to complications such as low birth weight, premature birth, low Apgar scores, shorter gestational ages, or even generating alterations in the prenatal bonding development [5,6]. Evidence suggests that a positive maternal attitude during pregnancy decreases the risk of adverse effects during the baby’s birth [7,8].

In order to comprehensively assess pregnant women’s mental well-being, it is necessary to emphasize that “mental illness” and “mental health” are different concepts, although closely related. In this sense, mental health involves the most objective possible analysis of thought processes, emotions, and behaviors, and its exploration requires identifying the extent to which individuals cope with adverse life experiences, how capable they are of establishing interpersonal relationships, and how they make appropriate decisions [9,10].

The concept of Positive Mental Health (PMH) was proposed by Marie Jahoda in 1958 [11]. This model is conceptualized from a mental health promotion perspective, focusing on implementing interventions to foster positive personal development [12]. This author defined six criteria for PMH: attitudes towards oneself, personal growth and self-actualization, integration, autonomy, perception of reality, and mastery of the environment.

Lluch subsequently developed the Multifactorial Model of Positive Mental Health (MMPMH, 1999), which focuses on developing a scale to measure how each person copes with situations that arise in their daily lives [12,13]. According to Lluch, PMH is defined as “a set of positive and negative feelings, thoughts, and behaviors; although it is important to foster states of well-being and happiness, it is also necessary to accept states of discomfort, understanding that often, what is truly mentally healthy—true positive mental health—is being sad, disappointed, or worried” [13].

Studies addressing the construct of PMH that have been conducted to date have focused primarily on the mental health of various populations [9,14,15], but, to our knowledge, only one recent study has addressed PMH in pregnant women [16]. Its findings showed that aspects like maternal age, basic education, anxiety, depression, and emotional loneliness were directly related to PMH. This connection between this construct and various sociodemographic and psychosocial variables is crucial, especially in the perinatal period [16].

Health services that care for pregnant women must give special attention to the various social determinants and risk factors that can influence women’s mental health, such as teenage pregnancy, poverty, gender discrimination, malnutrition, limited educational opportunities, physical health problems, no or limited social network, natural disasters, gender-based violence, unwanted pregnancy, infertility, and substance abuse [17,18,19].

Furthermore, pregnancy after the age of 35 is also a significant risk factor. Firstly, due to the difficulty of getting pregnant, which can cause increased stress and anxiety. And secondly, the risk of miscarriage is higher and there is a greater risk of premature birth [20,21,22].

However, there are protective factors that should be promoted, as they protect and foster perinatal mental health and are associated with fewer anxiety and depression symptoms during pregnancy. Some examples include factors such as strong social support, access to education, positive experiences in previous pregnancies, and high-quality healthcare during and after pregnancy [17,18].

On the other hand, greater maternal resilience has been linked to a lower risk of mental health problems during pregnancy and the postpartum period [18]. The age of the pregnant woman, her income, and her concerns during pregnancy negatively affect her resilience and capacity [20,21,22]. In this context, perinatal health is a period of significant change and vulnerability, in which mental healthcare is crucial for both the mother and the development of the baby [18,23]. It is important to note that emotional symptoms are one of the most studied areas in perinatal health. Stress during pregnancy has consequences for both the development of pregnancy and the subsequent development of the baby, which persists throughout the child’s life [18,23]. Research suggests the importance of PMH during pregnancy as a protective factor for children’s brain development [24,25].

It is worth mentioning that the prenatal bonding is emerging as a central and indispensable variable in perinatal health. Its development during pregnancy not only influences the immediate connection with the baby but it is also postulated as a predictor of the postpartum bond and, fundamentally, as a determining factor in the formation of the child’s character and personality in the adulthood [26,27].

The quality of the prenatal bonding can provide valuable information into the nature of the mother-infant relationship after birth.

Numerous studies have shown that the mother’s level of anxiety during pregnancy, stress, depression and unfavorable socio-economic factors are associated with the degree of prenatal bonding. In addition, it is important to mention the influence of social support on healthy pregnancy development. Importantly, the partner represents a key element in the development of prenatal bonding. Therefore, the care and promotion of prenatal bonding can enhance positive parenting practices and function as a protective factor for the postnatal relationship [27,28].

From a salutogenic perspective, it is of interest to understand the extent to which PMH can influence classic symptoms in perinatal health (such as anxiety, stress, or depression) while also promoting the development of a healthy bond. This integrative approach seeks not only to identify and mitigate risk factors, but also to enhance women’s resources and strengths, and promote fuller and healthier perinatal development.

**Figure 1:**
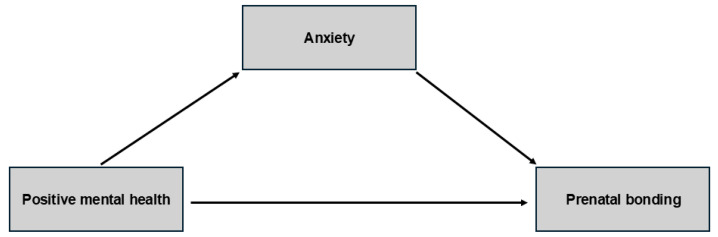
Proposed mediation model. The model examines the relationships among PMH, anxiety, and prenatal bonding, with anxiety serving as a potential mediator between PMH and prenatal bonding.

Keeping this in mind, the aim of the present study was to examine the psychological experiences of pregnant women, analyzing the factors that influence maternal well-being within the framework of the MMPMH. Specifically, the study aims to assess the extent to which PMH contributes not only to the modulation of classical mental health symptoms such as anxiety, but also to the promotion of the emotional bonding with the baby. As a result, we present the proposed mediation model (Figure 1), whose implications will be explained in detail in the results and discussion sections.

In addition, the study aims to address the following hypotheses:

**H1.**  *Mothers with better PMH are expected to experience stronger emotional connection with their babies.*

**H2.**  *Higher PMH is expected to be associated with lower anxiety during pregnancy.*

**H3.**  *Anxiety may influence prenatal bonding negatively, and is hypothesized to partially mediate the effect of PMH on that bond.*

**H4.**  *Socio-demographic variables (e.g., employment status, having a partner, etc.) are expected to be associated with both anxiety and prenatal bonding.*

## 2. Materials and Methods

A cross-sectional study was performed among 90 pregnant women aged 18 years or older who attended primary and specialist care centers during pregnancy follow-up visits. Participants were gradually incorporated into the study during sample collection through convenience sampling. The study received approval from the Ethics Committee of the Príncipe de Asturias University Hospital (code OE 07/2024) on 1 March 2024. Women’s consent was obtained prior to participation in the study.

### 2.1. Participants

The inclusion criteria considered in this study were pregnant women of legal age with a good command of spoken and written Spanish. The exclusion criteria for the study were pregnant women diagnosed with a current serious psychiatric disorder who were admitted to an acute care ward or who had an intellectual disability. The sample was obtained by contacting midwives and nurses from different health centers. Associations such as the Spanish Midwives Association were also contacted. The questionnaires were distributed through social media and at strategic locations.

### 2.2. Outcome Measures

For this study, an ad hoc online questionnaire was developed. The first part of the questionnaire included self-reported data on socio-demographic variables such as age, marital status, educational level, and employment status. Relevant clinical information on obstetric history and data on the current pregnancy was also collected.

The second part of the questionnaire assessed psychological variables such as The Abbreviated Positive Mental Health Questionnaire, that was developed from the Positive Mental Health Questionnaire originally created by Lluch in 1999 [12]. The original version had 39 items, but this brief self-administered version consists of 18 items that assess six factors: personal satisfaction (F1), prosocial attitude (F2), self-control (F3), autonomy (F4), problem solving and self-actualization (F5), and interpersonal relationship skills (F6). Each item is scored on a Likert scale ranging from 1 to 4: always or almost always, very often, sometimes, never or almost never. The higher the score, the greater the PMH. The questionnaire has good psychometric properties, with a reliability of 0.88 and an internal consistency coefficient between 0.72 and 0.79 [29].

To assess the emotional bond between pregnant woman and unborn child, the Prenatal Assessment Scale for Pregnant Women (EVAP) was used, initially designed by Lafuente in 2008 [30] and subsequently validated by Artica-Martínez et al. in 2018 [31], forming a structure with 21 items and two dimensions: adaptation to pregnancy (12 items) and prenatal affective bonding (9 items). In terms of the reliability of the scale, values of 0.746 and 0.749 were obtained for these dimensions, respectively. Finally, the Hospital Anxiety and Depression Scale (HADS; Zigmond & Snaith, 1983) [32] was used to measure anxiety symptoms in pregnant women. The HADS is a self-administered screening instrument composed of 14 items, divided equally into two subscales: anxiety (7 items) and depression (7 items). Each item is rated on a 4-point Likert scale ranging from 0 to 3, allowing for a total score between 0 and 21 for each subscale. The anxiety subscale, which was the focus of this study, has demonstrated high internal consistency, with a Cronbach’s alpha of 0.86. The HADS has been extensively validated, also in Spanish, and is widely used in both clinical and non-clinical settings, showing strong psychometric properties across diverse adult populations, including pregnant women [33].

### 2.3. Statistical Analysis

Data analysis was carried out using IBM SPSS Statistics version 27.0 [34]. Descriptive statistics were computed for all variables, encompassing frequencies and summary measures such as mean, standard deviation, minimum, and maximum values. These preliminary analyses offered insight into the overall distribution and central tendencies of the dataset. To identify potential covariates, a range of inferential statistical procedures was applied. Independent samples t-tests were utilized to compare group means, while one-way ANOVA was conducted to detect differences across three or more groups. Associations between categorical variables were examined using chi-square tests. Pearson correlation coefficients were calculated to investigate possible covariates and to assess inter-variable relationships within the proposed model, serving as a prerequisite for mediation analysis.

To examine more complex relational patterns, mediation analyses were performed using the PROCESS macro for SPSS [35]. A basic mediation model (Model 4) was employed to evaluate whether the impact of the predictor (PMH) on the outcome variable (prenatal bonding) was mediated by a third factor (anxiety). To test the significance of indirect effects in model, bootstrapping—a non-parametric resampling technique—was implemented. This method involves generating a distribution of the indirect effect by repeatedly resampling the data (typically between 5000 and 10,000 iterations), enabling the estimation of confidence intervals. Indirect effects were considered statistically significant when the 95% confidence intervals excluded zero [35]. All statistical tests were conducted with a significance threshold set at *p* < 0.05.

## 3. Results

### 3.1. Descriptive Data

Table 1 shows participants’ sociodemographic, obstetric and clinical data. A total of 90 women with an average age of 34.25 (SD = 5.05) constituted the sample of this study. Most women were married or living with a partner (90%, n = 81). All women had formal education, with 57.8% holding a university degree (n = 52). Most women were employed (85.6%), of whom 68.9% worked full-time and 71.1% (n = 64) had a permanent contract.

Regarding previous obstetric data, 56.6% (n = 51) of women were primiparous, and 18.9% (n = 17) have had a previous miscarriage. About 12.2% (n = 11) had a higher-risk pregnancy and 18.9% (n = 17) had become pregnant through assisted reproduction techniques.

Concerning anxiety levels and according to the cut-off points established by Zigmond & Snaith [32], 72.6% fell within the normal range (scores 0–7), 17.9% were classified as borderline cases (scores 8–10), and 9.5% exceeded the clinical threshold for severe anxiety (scores ≥ 11).

### 3.2. Descriptive Statistics and Correlations Between Variables of Interest

Table 2 shows the descriptive statistics and correlations among the variables involved in the model. As can be seen in Table 2, there are significant negative correlations between anxiety and PMH (*p* < 0.001), and between anxiety and prenatal bonding *(p* = 0.047). PMH maintains significant and positive correlations with prenatal bonding (*p* < 0.001).

### 3.3. Analysis of Possible Covariates

In order to control for potential confounding factors, different sociodemographic and obstetric variables were proposed as possible covariates to include in the mediation model. Specifically, the following covariates were considered: age, education level, having a partner, previous abortions, previous births, risky pregnancy, assisted reproduction techniques. Maternal age has been associated with psychological outcomes during pregnancy [36], while education level is consistently linked to maternal and perinatal health [37]. Partner support plays a crucial role in perinatal mental health [38]. Previous abortions have been examined in relation to maternal psychological outcomes [39], and parity has been identified as a relevant factor in maternal mental health [40]. Risky pregnancies are known to increase prenatal stress [41], and assisted reproduction techniques have been associated with perinatal emotional well-being [42]. Including these covariates enhances the robustness of the analyses and provides greater transparency regarding their influence on the mediation pathways.

With the aim of including in the model only the covariates that were statistically significant in relation to the variables of the proposed mediation model (PMH, anxiety, and prenatal bonding), the corresponding bivariate analyses were carried out.

Table 3 shows the statistically significant differences obtained between the considered covariates and the variables included in the model. Table 4 presents the mean scores and standard deviations for each of the subgroups (yes/no) in the covariates (i.e., working, having a partner, previous abortions, risk pregnancies) that were found to be significant with respect to some of the variables considered in the model (PMH, anxiety and prenatal bonding) (Table 3). Statistically significant differences were observed with respect to having a partner and prenatal bonding (t = −2.603; *p* = 0.03) and PMH (t = −3.117; *p* = 0.01), with higher scores observed in both cases among women with partners. On the other hand, working maintained significant relationships with prenatal bonding (t = −2.331; *p* = 0.028), with lower scores observed among working women.

Significant differences in previous abortions were also observed with respect to anxiety (t = −2.826; *p* = 0.006) and PMH (t = 2.454; *p* = 0.016), with higher and lower scores, respectively, in women who have suffered previous abortions.

Finally, having a risky pregnancy had significant relationships with PMH (t = −2.270; *p* = 0.035), with lower scores observed in women with high-risk pregnancies.

No other significant relationships were observed with respect to the possible covariates considered.

### 3.4. Theoretical Proposed Model

Table 5 presents the findings from simple mediation analysis (PMH, anxiety and prenatal bonding). Based on the covariate assessment conducted in the previous section (see Table 3 and Table 4), employment status, cohabitation with a partner, history of previous abortions, and high-risk pregnancy were identified as relevant covariates to be included in the model.

In particular, the model posited anxiety as a mediating variable between PMH and prenatal bonding. The results showed that PMH is associated, in the proposed model, with prenatal bonding (Total effect of X on Y: Effect= 0.683, SE = 0.081, t = 8.356, *p* < 0.001, 95% CI = 0.520, 0.845). There was also a significant direct effect between PMH and prenatal bonding (Effect= 0.777, SE = 0.09, t = 8.556, *p* < 0.001, 95% CI = 0.596, 0.958). The indirect effect of PMH on prenatal bonding through anxiety was not statistically significant (Effect= −0.094, SE= 0.068, 95% CI = −0.252, 0.008). The proposed model contributed to the explanation of 56% of the variance of prenatal bonding (F = 16.617, *p* < 0.001). Specifically, PMH was negatively related to anxiety (a = −0.297, SE = 0.062, t = −4.784, *p*< 0.001, 95% CI =−0.420, −0.173). On the other hand, anxiety influenced prenatal bonding (b = 0.316, SE = 0.145, t = 2.174, *p* = 0.032, 95% CI =0.026, 0.606).

**Figure 2:**
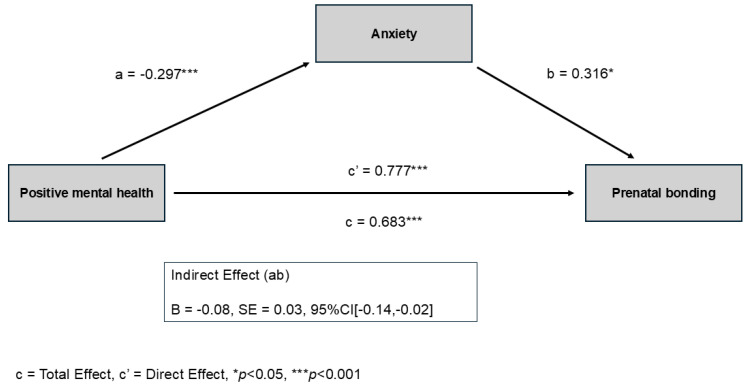
Coefficients of the proposed simple mediation model. Positive mental health (PMH) predicts lower anxiety (path a) and higher prenatal bonding both directly (path c′) and indirectly through anxiety. Anxiety is positively associated with prenatal bonding (path b), producing an indirect effect in the opposite direction to the direct effect. This indicates partial mediation, meaning that PMH is related to prenatal bonding both directly and through anxiety. * *p* < 0.05; *** *p* < 0.001.

Regarding the role of covariates, significant effects of working on prenatal bonding (Effect =−2.806, SE = 1.226, t = −2.287, *p*= 0.024, 95% CI =−0.363, −5.248) was observed, indicating that, after accounting for the variables included in the model (see Table 4), employed women reported lower scores in prenatal bonding. Figure 2 showed the coefficients of the relationships between the variables involved in the model.

## 4. Discussion

The aim of the present study was to examine mental well-being during pregnancy through the lens of the PMH model, an approach that has received limited attention in the literature to date. The findings are consistent with the theoretical framework and conceptual definitions of PMH. Specifically, PMH was positively associated with prenatal bonding both directly and indirectly through anxiety, which partially mediated this relationship. Higher PMH was related to lower anxiety, and anxiety in turn was associated with increased prenatal bonding. The study also provides novel insights by evidencing associations between mental well-being and specific sociodemographic variables of pregnant women, such as working status, as well as their perceived quality of prenatal bonding. Most existing studies have concentrated on postnatal maternal-infant bonding, with far fewer addressing prenatal bonding during pregnancy, as examined in our study.

Previous research has determined the relationship between the mother’s psychological well-being and prenatal bonding, establishing that the stronger the bond, the greater its protective effect against perinatal anxiety and depression [43,44]. This association can extend up to the first year after birth and to the mother’s perception of the child’s temperament [45,46]. In this regard, our results have also shown the existence of a negative relationship between prenatal bonding and anxiety, as well as a positive relationship between prenatal bonding and PMH. Therefore, promoting the development of PMH and a strong, high-quality prenatal bonding can protect not only the mental health of the pregnant woman but also the well-being of children in the early stages of life [44,47,48].

With regard to the results of our simple mediation model, we observed a direct effect of PMH on the formation of the prenatal bonding, regardless of anxiety levels. Furthermore, we observed that higher PMH contributes to lower anxiety levels. In addition, anxiety partially mediated the relationship between PMH and prenatal bonding, such that higher anxiety was associated with stronger prenatal bonding along this indirect pathway. These findings are innovative and challenge the initially proposed hypothesis regarding the consistently negative role that anxiety might play in this modulation.

One possible explanation for this unexpected pattern may be understood in terms of adaptive or maladaptive emotionality. In other words, anxiety that is attenuated and redirected by a prior basis of PMH can become “adaptive anxiety”, reinforcing the bond with the baby. Previous studies indicate that experiencing some anxiety during pregnancy is common; the challenge is to manage it through adaptive strategies. Furthermore, it is difficult to assess the threshold at which anxiety becomes pathological, and there is a need for assessment tools specifically adapted to this population [49].

Therefore, assessing other dimensions from a salutogenic perspective, such as PMH, can help predict both the risks and strengths that are needed to be developed in pregnant women [16,50]. Existing research shows that the pregnancy itself is associated with varying levels of anxiety at different stages; however, anxiety becomes a challenge when it interferes with a woman’s daily life. Pregnant women who experience mild, time-limited anxiety are able to focus their attention on tasks that require concentration. However, when anxiety levels are high, they consume a significant amount of a woman’s time and interfere with her ability to fulfill her responsibilities, maintain her relationships, care for herself, and relax [51]. Severe cases of anxiety may be associated with behavioral consequences, such as negative attitudes and an excessive search for tranquility, as well as evasive behaviors that could diminish the prenatal bonding [52,53].

Moreover, pregnancy is a period of constant adaptation to stressors for women. Recent evidence supports a multi-level view of prenatal anxiety, suggesting that it is not always a sign of pathology but may also reflect an adaptive recalibration of the maternal brain, psychology, and caregiving system. Neurobiological studies show that the transition to motherhood involves hormone-driven changes in the brain, especially in regions related to emotional processing, social understanding, and caregiving behavior [54,55]. These changes may help prepare women for the demands of caring for a newborn. At the same time, psychological research highlights the central role of emotion regulation during pregnancy: as noted by Penner and [56], the way pregnant women manage their emotions can determine whether anxiety and increased alertness function in an adaptive way or become sources of stress and mental health problems. Longitudinal findings also show that both general anxiety and pregnancy-specific anxiety tend to decrease as pregnancy progresses, suggesting that many women naturally adjust to the emotional challenges of this period [57]. Interestingly, this study also indicate that secure attachment does not necessarily predict lower levels of this type of anxiety, possibly because situational factors (i.e., attending medical appointments or undergoing diagnostic tests), can temporarily increase anxiety even in otherwise secure women. However, other studies remind us that high or poorly regulated prenatal anxiety can have negative effects. For example, greater anxiety during pregnancy, especially in women with lower self-regulation, has been linked to more unpredictable maternal signals during early interactions with the baby, which may affect caregiving quality [58]. Together, these findings suggest that the neurobiological plasticity of pregnancy may operate as a “double-edged sword”: it can support adaptive responses that enhance vigilance and maternal sensitivity, but it may also increase vulnerability to mental health problems when risk factors such as insecure bonding/attachment, adverse experiences, or limited coping resources are present. This interpretation is also reflected in our findings, which show that higher prenatal anxiety was associated with greater maternal sensitivity and communication with the unborn child, supporting the idea that regulated anxiety may serve as an adaptive function. Ultimately, advancing this line of research will require distinguishing between types of anxiety, attachment patterns, and stress reactions mediated by coping strategies, as this would help identify risk profiles (including insecure attachment or maladaptive coping) that may hinder this adaptive process.

Recent research also aligns with our findings regarding factors related to a history of previous miscarriages or high-risky pregnancies. Several determinants have been associated with prenatal depression and anxiety, including the absence of a partner or limited social support, previous experiences of abuse or intimate partner violence, a history of mental health disorders, unplanned or unwanted pregnancies, exposure to adverse life events and high perceived stress, as well as current or past pregnancy complications and experiences of pregnancy loss [59]. Studies that have assessed high-risky pregnancies following fertility treatments have determined that in most couples, the level of prenatal bonding was similar to or higher than that of couples who conceived without assisted reproductive technology [60]. However, couples undergoing these treatments may be more susceptible to anxiety due to pregnancy loss; therefore, support could focus more on pregnancy-related anxiety in these couples than on any attachment/bonding-related intervention [61]. Similarly, a systematic review revealed that women who conceived through fertility treatments reported lower social and physical functioning, as well as higher levels of anxiety and depression, compared to women who conceived spontaneously. The authors also showed women difficulties in adjusting to pregnancy and experienced discontinuities in care, both between discharge from the fertility clinic and the first visit to local maternal care services, and in postnatal care [62].

With respect to the presence of previous miscarriages, this factor has been associated with higher levels of anxiety. The studies reviewed identify prior pregnancy loss as a significant risk factor for women’s mental health [63,64]. A systematic review reported that levels of anxiety and depression following perinatal loss are significantly higher compared to “no loss” controls (i.e., live births, non-pregnant women, or those with difficult live births). Higher rates of depression and anxiety have also been observed in women who experienced pregnancy loss during the later stages of gestation [65].

Our study is innovative because, in addition to evaluating the correlation between miscarriages and anxiety, we also evaluated their relationship with PMH. Most research focuses solely on the negative aspects of mental health, and very few assess the positive dimensions or strengths within this population. However, the limited studies that have explored aspects related to PMH have exhibited that women with previous miscarriages have lower levels of spiritual intelligence and eudaimonia, and that the presence of anxiety is also related to maladaptive coping strategies [66,67,68]. It is therefore essential to consider this and other risk factors to identify women who may require additional support and to promote interventions aimed at preventing mental health problems and promoting effective coping strategies. This need is especially relevant when psychological management is not routinely addressed in health services, and where standardized protocols, including grief training for healthcare professionals, are still lacking [69].

Among the protective factors for maternal mental health, social support, specifically partner support, plays a critical role. Evidence has demonstrated that pregnant women who perceive higher levels of social support report lower levels of depression and anxiety, as well as greater coping capacity and self-efficacy [70,71,72,73]. These findings are consistent with the results of our study, in which women with a stable partner exhibited higher levels of PMH. The impact of the partner relationship also extends to the development of prenatal bonding, partly through its influence on maternal stress and anxiety, and partly through its effect on coparenting dynamics. However, studies examining the role of partner support in shaping prenatal bonding have yielded inconclusive results. Some investigations suggest no association between coparenting and weaker bonding, while others report a positive relationship, which is consistent with the findings of our study. Nevertheless, the literature supports that coparenting exerts a protective effect against maternal anxiety and depression in the postpartum period, which indirectly contributes to the quality of prenatal bonding [74,75].

Previous research indicates that exposure to stress and stressful life events can adversely affect the prenatal bonding process. Consequently, it is essential to identify the specific factors that contribute to heightened stress and may compromise the quality of this bond across different stages of pregnancy and the postpartum stage. Studies investigating these determinants have suggested that sociodemographic characteristics, including employment status, may act as sources of maternal stress. Although some evidence has indicated that mothers with stable employment are at lower risk of experiencing difficulties in prenatal bonding compared to unemployed mothers, other research has been inconclusive. In fact, our findings diverge from this trend [76,77]. In our sample, despite the majority of mothers were employed, employment status was associated with lower prenatal bonding scores compared to those who were not working. However, it is not possible to determine whether the difference in conclusions may be explained by working conditions, job security, or the broader family economic resources available beyond the mother’s individual employment income. It can therefore be inferred that maternal employment status exhibits a complex and heterogeneous association with prenatal bonding, whereby outcomes may be either beneficial or detrimental depending on contextual factors such as the timing of employment, work–life balance, and broader socioeconomic conditions.

In this regard, *The State of Motherhood in Europe* 2024 survey, conducted with 9600 mothers across 12 European countries, highlights the following findings: 57% of Spanish mothers report mental health problems (compared to a European average of 50%); 42% report anxiety (compared to a European average of 32%); and 21% suffer from exhaustion/burnout (compared to a European average of 18%). The survey also shows that Spanish mothers continue to take on up to 64% of domestic tasks, regardless of their employment status, with significant consequences for their well-being, career, and financial stability: after the birth of their first child, the proportion of mothers working full-time drops from 79% to 52%; 6% leave the labor market altogether, one in three feels dissatisfied with their professional situation; and 53% feel that their role is not recognized by society (compared to a European average of 59%) [78]. Recent national data also indicate uneven access to parental leave in Spain, and although leave equalization has progressed, many mothers continue to face reduced employment prospects and domestic overload [79,80]. Meanwhile, reviews show that paid parental leave is associated with better early mother-child care and may strengthen emotional bonds, by affording parents more time for attentive, responsive caregiving in the critical first months [81,82]. Within this structural context, that is, limited leave, unequal domestic burden, or labor-market penalties for motherhood, the anticipation of these challenges may already shape the prenatal bonding during pregnancy, returning to work shortly after birth may reduce opportunities for sensitive bonding, potentially explaining the lower scores observed among employed mothers in our sample.

Building on this, international evidence further supports the need for policies that begin supporting mothers during pregnancy rather than only after birth. Prenatal anxiety and depression are common and predict adverse perinatal outcomes and modifiable stressors such as poor support or work overload are key risk factors, underscoring the need for preventive measures during pregnancy [59]. Longer paid maternity leave has been linked to reductions in neonatal and infant mortality and improvements in early childhood health, indicating that leave with income protection offers benefits beyond the immediate postpartum period [83]. Similarly, research on prenatal bonding highlights that the quality of the mother’s prenatal bonding, predicts her postnatal bonding with the infant, and maternal well-being, reinforcing the idea of providing support prior to childbirth to optimize dyadic outcomes [84]. International standards (International Labour Organization-ILO Convention No. 183) recommend at least 14 weeks of maternity leave, with a target of 18 weeks in Recommendation No. 191; many Organization for Economic Cooperation and Development (OECD) countries exceed these minimum levels [85]. The Nordic models offer an illustrative comparison: Sweden provides 480 days of paid parental leave with high wage replacement and flexible use (e.g., transferable days, even to grandparents), and Iceland and Norway maintain similarly generous gender-balanced schemes beginning already during the perinatal period. In contrast, Spain has achieved equal and fully paid leave for both parents (recently extended) (Royal Decree-Law 9/2025) [86], which represents a significant step toward parity, but still remains considerably shorter and less flexible that the Nordic Systems. Beyond leave duration, pregnancy-friendly workplace accommodations, such as flexible hours, adaptations of physical demands, or protected time for prenatal appointments, are consistent with the epidemiology of prenatal mental health and the biology of the prenatal bonding, likely reducing stress exposure and improving maternal-child outcomes. In summary, policies that shift support to pregnancy, through extended paid leave and viable workplace adjustments, are aligned with global standards and the best available evidence linking prenatal conditions to maternal mental health and early childhood health [87].

Additionally, pregnancy, postpartum, and early parenthood provide special opportunities to develop skills that create family environments conducive to healthy development. This involves emphasizing the importance of fostering prenatal bonding, strengthening couple relationships, cultivating social support networks, and safeguarding the mother’s physical and mental health [88]. Although evidence on specific programs promoting PMH in pregnant women remains limited, existing studies report positive results, including reductions in anxiety, stress, and depression [89,90].

Healthcare professionals, particularly nurses and midwives, play a key role in promoting maternal–baby health by integrating psychosocial support and mental health assessment into routine antenatal care. Their close and continuous contact with pregnant women places them in a unique position to identify early signs of psychological distress, provide counseling, and reinforce coping strategies that reduce the risk of anxiety and depression. Scientific evidence shows that midwife-led continuity models are associated with greater maternal satisfaction, lower intervention rates, and better psychosocial outcomes, highlighting the importance of incorporating mental health promotion into their practice [91]. Furthermore, specific interventions led by nurses and midwives, such as structured antenatal education, stress management programs, and relationship-focused counseling, have been shown to strengthen prenatal bonding, which in turn predicts a more positive postnatal bond and caregiving behaviors [92]. Their dual focus on physical and psychological well-being makes them essential actors in translating policy measures, such as extended maternity protection and workplace accommodations, into tangible health benefits for mothers and babies, fostering supportive environments and ensuring that women have access to informational and emotional management resources. In this sense, the integration of nurse- and midwife-led interventions into maternal health systems can be considered an indispensable complement to structural policy reforms, as it ensures the promotion of mental health and prenatal bonding from the initial stages of pregnancy [93].

## 5. Limitations

Several limitations should be noted. First, the cross-sectional design prevents causal inferences about the observed associations. Second, the relatively small sample size may limit statistical power and generalizability; moreover, the absence of an a priori power analysis further constrains the interpretation of the findings. Nevertheless, methodological guidelines indicate that mediation analyses with medium effects can be adequately conducted with samples of this magnitude [94]. Third, all assessments relied exclusively on self-report instruments, which may introduce response biases and limit the objectivity of the measures obtained. In addition, parental bonding is inherently a prenatal construct and therefore reflects only the mother’s disposition during pregnancy, without capturing the actual mother–infant bond that will later develop. As a result, any implications for postpartum bonding or newborn health should be interpreted with caution, as they remain speculative. Furthermore, the absence of a clinical psychological evaluation may also constitute a source of bias, as undetected psychopathological symptoms could influence both PMH and prenatal bonding reports. Finally, participants were recruited from various institutions in Madrid, so the sample may not be fully representative of the general population of pregnant women. Therefore, future research should include larger and more diverse samples to validate and extend these findings.

## 6. Conclusions

This study underscores the role of PMH as a psychological strength during pregnancy. Higher PMH levels were associated with reduced anxiety and stronger prenatal bonding, with anxiety partially mediating this relationship. Importantly, a modest level of “adaptive” anxiety embedded within a positive mental framework appeared to enhance attentiveness and emotional connection to the unborn child. Demographic and obstetric factors, such as having a stable partner, employment status, and history of miscarriage or high-risk pregnancy, were also linked to PMH, anxiety, and prenatal bonding, indicating the complexity of contextual influences.

The findings call for a salutogenic focus on perinatal care that strengthens psychological resources rather than solely addressing risk factors. Policies should ensure adequate maternity leave, financial support, and work–life balance provisions to foster maternal mental health throughout pregnancy and the postpartum period. Lastly, nurses and midwives are pivotal in integrating mental health promotion into antenatal services; through early screening, psychosocial support, and bonding-enhancing interventions, they can help translate these insights into improved maternal and infant outcomes.

## Figures and Tables

**Table 1:** Sociodemographic, obstetric and clinical data.

Characteristics	n	%	Mean	SD	Maximum	Minimum
Age			34.25	5.05	44	19
Gestational week			25.69	9.7	41	3
Relationship status						
Living with partner	81	90				
Living without partner	9	10				
Educational level						
Primary education	9	10				
Secondary education	16	17.8				
University education	52	57.8				
Employment situation						
Full-time	62	68.9				
Part-time	15	16.7				
Unemployed	9	10				
Domestic work/childcare	4	4.4				
Risky pregnancy	11	12.2				
Assisted reproductive techniques	17	18.9				
Previous pregnancies						
None	51	56.6				
One	35	38.8				
Two	4	4.4				
Previous miscarriage	17	18.9				
Anxiety scores (HADS)						
Normal range (score 0–7)	65	72.6				
Borderline cases (score 8–10)	16	17.9				
Severe anxiety (score ≥ 11)	9	9.5				

SD: standard deviation.

**Table 2:** Descriptive data and correlations between variables of interest.

		Mean	SD	Range	Asy.	Kurt.	2.	3.
1. Positive mental health		51.36	5.60	37–61	−0.50	−0.39	−0.52 **	0.68 **
2. Anxiety		6.26	3.45	0–19	1.02	2.01		−0.21 *
3. Prenatal bonding		36.21	5.55	16–44	−1.24	2.04		

** *p* < 0.01; * *p* < 0.05; Asy: Asymmetry; Kurt: Kurtosis.

**Table 3:** Analysis of possible covariates (Significant findings from bivariate analyses of mean differences).

	Positive Mental Health	Anxiety	Prenatal Bonding
Working (yes/no)	ns	ns	*p* = 0.028
Having a partner (yes/no)	*p* = 0.01	ns	*p* = 0.03
Previous abortions (yes/no)	*p* = 0.006	*p* = 0.016	ns
Risky pregnancies (yes/no)	*p* = 0.035	ns	ns

ns: non-statistically significant mean differences; *p*: *p*-value for statistically significant mean differences.

**Table 4:** Statistically significant differences between the considered covariates and the variables included in the model.

	Having a Partner		Working		Previous Abortions		Risky Pregnancy	
	Yes	No	Yes	No	Yes	No	Yes	No
	Mean (SD)	Mean (SD)	Mean (SD)	Mean (SD)	Mean (SD)	Mean (SD)	Mean (SD)	Mean (SD)
Positive Mental Health	51.87 (5.41)	44.20 (3.63)			48.85 5.81	52.20 (5.35)	48.90 (3.47)	51.70 (5.77)
Anxiety					8.22 (4.41)	5.73 (2.96)		
Prenatal bonding	36.49 (5.41)	29.25 (5.62)	35.77 (5.74)	38.62 (3.64)				

SD: standard deviation.

**Table 5:** Simple mediation model: Effects of positive mental health on prenatal bonding through anxiety (Model 4).

DV: Anxiety	R^2^	F	*p*	Beta	SE	t	*p*	LLCI	ULCI
Model summary	0.31	7.063	<0.001						
IV: PMH				−0.297	0.062	−4.784	<0.001	−0.420	−0.173
Abortions (covariate)				1.522	0.829	1.836	0.070	−0.128	3.173
With a partner (covariate)				0.115	1.092	0.105	0.916	−2.059	2.290
Risky pregnancy (covariate)				1.428	1.064	1.342	0.183	−0.690	3.548
Working (covariate)				0.877	0.932	0.941	0.349	−0.978	2.733
DV: Baby bonding									
Model summary	0.56	16.617	<0.001						
IV: PMH				0.777	0.091	8.556	<0.001	0.596	0.958
M: Anxiety				0.316	0.145	2.179	0.032	0.026	0.606
Abortions (covariate)				0.151	1.088	0.139	0.889	−2.016	2.319
With a partner (covariate)				1.952	1.405	1.389	0.168	−0.844	4.750
Risky pregnancy (covariate)				−2.530	1.384	−1.828	0.071	−5.287	0.226
Working (covariate)				2.528	1.205	2.097	0.039	0.128	4.928

DV: Dependent variable; IV: Independent variable; LLCI: Lower Limit Confidence Interval; ULCI: Upper Limit Confidence Interval; M: Mediator; PMH: Positive mental health.

## Data Availability

The data will be made available upon request to the corresponding author. The datasets generated and/or analyzed during the current study have not been deposited to a public repository due to privacy and ethical restrictions. The data include sensitive clinical and socio-demographic information from pregnant women, which could potentially compromise participant confidentiality even after anonymization, as certain combinations of variables might allow the eventual identification of individual participants. According to the approval granted by the institutional ethics committee, data sharing is limited to requests that ensure compliance with data protection regulations and ethical standards. A minimal data set supporting the main findings (including aggregated or de-identified variables relevant to the statistical analyses) will be made available upon reasonable request to the corresponding author, provided that the requester meets the ethical and legal requirements for access to confidential data.

## Abbreviations

The following abbreviations are used in this manuscript:

PMH	Positive Mental Health
OECD	Organization for Economic Cooperation and Development
